# Patient factors associated with waiting time to pediatric rheumatologist consultation for patients with juvenile idiopathic arthritis

**DOI:** 10.1186/s12969-020-0413-7

**Published:** 2020-03-06

**Authors:** Claire E. H. Barber, Cheryl Barnabe, Susanne Benseler, Ricky Chin, Nicole Johnson, Nadia Luca, Paivi Miettunen, Marinka Twilt, Dwaraka Veeramreddy, Natalie J. Shiff, Heinrike Schmeling

**Affiliations:** 1 Division of Rheumatology, Department of Medicine, Cumming School of Medicine, University of Calgary, 3280, Hospital Dr NW, Calgary, AB T2N 4Z6 Canada; 2 Arthritis Research Canada, 5591 No 3 Rd, Richmond, BC V6X 2C7 Canada; 3 Alberta Children’s Hospital, Department of Pediatrics, Cumming School of Medicine, University of Calgary, 28 Oki Drive, NW, Calgary, AB T3B-6A8 Canada; 4 Alberta Children’s Hospital Research Institute, Heritage Medical Research Building, 3330 Hospital Dr. NW, Calgary, AB T2N 4N1 Canada; 5 Department of Community Health and Epidemiology, University of Saskatchewan (adjunct), Health Science Building, 107 Wiggins Road, Saskatoon, SK S7N 5E5 Canada

**Keywords:** Arthritis, Juvenile, Quality of health care, Wait times

## Abstract

**Background:**

Early diagnosis and treatment of Juvenile Idiopathic Arthritis (JIA) is essential to optimize outcomes. Wait times (WTs) to consultation with a pediatric rheumatologist consultation is a Canadian quality measure, with benchmarks set at 7 days for systemic JIA (sJIA) and 4 weeks for other JIA categories. In this study we assess WTs for JIA at a single academic center and describe factors associated with longer WTs.

**Methods:**

This was a retrospective cohort study of 164 patients enrolled in a pharmacogenetic study in Alberta between 2002 and 2018. Limited chart reviews were conducted to evaluate dates of referral and first rheumatology visit to calculate WTs for receipt of pediatric rheumatology care. Cox proportional hazard models identified factors associated with WTs considering variables at the first pediatric rheumatology visit including: JIA category, age, sex, distance to the pediatric rheumatology clinic, number of active joints, pain and C-reactive protein.

**Results:**

The median age at diagnosis was 8.0 years (interquartile range, IQR 3.5, 12.0) and 46% of patients had oligoarticular JIA. Only 18 patients (11%) were from rural locations. The median WT for all patients met the national benchmark (22 days, IQR, 9, 44) with no statistically significant difference between WTs among JIA categories (*p* = 0.055). Importantly, the majority of sJIA cases met the 7-day benchmark (67%) with a median WT of 1.5 days. Older age was associated with longer WT (HR 0.94, 95% CI 0.89, 0.98, *p* = 0.005).

**Conclusion:**

Median benchmarks were met, however delays in older patients highlight the need for monitoring WTs.

## Background

Juvenile idiopathic arthritis (JIA) is a chronic inflammatory condition affecting approximately 0.11% of females and 0.07% of males younger than 16 years of age in Canada [1, 2]. Timely diagnosis and treatment of JIA is important to achieve optimal patient outcomes [3]. Wait time (WT) to pediatric rheumatology care for patients with JIA is a nationally endorsed quality measure in Canada, with benchmark times from referral to pediatric rheumatology care of 7 days for systemic JIA (sJIA), and 4 weeks for other JIA categories [4, 5]. The purpose of this study was to measure and evaluate patient factors associated with WTs at a single academic pediatric rheumatology center.

## Materials & methods

### Data sources

This was a retrospective cohort study of JIA patients at a tertiary pediatric center in Calgary, Alberta, Canada who were participating in a pharmacogenetics study, for which all confirmed JIA cases at the center had been eligible for recruitment. Included patients had demographic and disease-related data collected prospectively between 2002 and 2018. As the pharmacogenetics study did not assess WTs, a further limited chart review was conducted to extract the dates of referral and first rheumatology visit, in order to calculate WTs. Referrals are received centrally and triaged by a physician within 1–3 days and categorized as urgent, semi-urgent or routine. The target wait times for urgent referrals are 1 week, for semi-urgent 4–6 weeks and no specific benchmarks are set for routine referrals.

### Variables and outcomes

Patients were classified into JIA categories based on the International League of Associations for Rheumatology Classification Criteria for JIA (ILAR) criteria at baseline [6, 7]. The primary outcome was the percentage of JIA patients seen within the benchmark WTs: 7 days for sJIA and 4 weeks (28 days) for all other JIA categories [4]. Factors evaluated for their association with WTs included JIA category, age, sex, distance from residence to the pediatric rheumatology clinic, number of active joints, pain (measured with a 10 cm visual analog scale, VAS) and C-reactive protein (CRP) at the first pediatric rheumatology clinic visit. Distance from clinic was calculated using the network method, which involves geocoding the latitude and longitude of the patient’s postal code using the Postal Code Conversion File, with distance calculations made using ArcGIS by calculating the shortest distance to the clinic using roadways [8]. Residences were classified as urban or rural according to Statistics Canada definitions, where urban areas were defined as areas with a minimum of 1000 population and a minimum population density of 400 persons per square kilometer (Km) [9].

### Data analysis

Descriptive characteristics and WT measures were reported using medians (interquartile range, IQR) given non-normal distributions. Non-parametric Kruskal-Wallis rank test was used to examine difference in WTs among JIA categories. Cox proportional hazard modeling was used to evaluate factors associated with WTs. Hazard ratios (HR) and 95% confidence intervals (95% CI) were estimated. Factors included in the model were based on clinical relevance and data availability. The base model included age at diagnosis, sex, network distance to center, number of active joints, baseline pain VAS score and CRP. Rheumatoid factor (RF) was excluded from the model due to collinearity with active joint count. A sensitivity analysis was conducted using a second model, which included age at diagnosis, sex, network distance to the center, JIA category and pain score. The proportionality assumption for the models was tested using the Schoenfeld test. Analysis was conducted using Stata 13.1.

### Ethics approval

All patients in the study provided written informed consent and the study was approved by the University of Calgary Research Ethics Board (REB15–0998).

## Results

### Patient characteristics

Baseline demographic characteristics are shown in Table 1. Of the 336 JIA patients enrolled in the pharmacogenetics study, only 164 had referral dates available and could be included in the study. One hundred and sixteen were female (71%), and the median age at diagnosis was 8.0 years (IQR 3.5, 12.0). The majority of patients had oligoarticular JIA (*n* = 75, 46%) or RF negative polyarticular JIA (*n* = 48, 29%), and 6 patients had sJIA (4%). The majority (*n* = 102, 62%) were from Calgary, and only 18 patients (11%) lived in rural locations. The median network distance for patients between their residence and the clinic was 22.8 Km (IQR 13.5, 127.8).

**Table 1:** Baseline patient characteristics

Characteristic	Number (%) or Median (IQR)
Demographics & Patient Location	
Female	116 (70.7)
Age at diagnosis	8.0 (3.5, 12.0)
Caucasian Ethnicity	118 (72.0)
Calgary (within city limits)	102 (62.2)
Outside of Calgary (outside of city limits)	62 (37.8)
Rural	18 (11.0)
Urban	146 (89.0)
Network Distance to pediatric center (Km)	22.8 (13.5, 127.8)
Baseline Disease Characteristics	
Number of Patients with > 4 Swollen Joints, *n* = 160	55 (34.4)
Median Number of Swollen joints, *n* = 160	2.0 (1.0, 6.0)
Number of Patients with > 4 ^a^Active Joints, *n* = 160	58 (36.3)
Median Number of ^a^Active joints, *n* = 160	2.0 (1.0, 6.0)
Pain VAS (0–100)	37.0 (19.0, 64.0)
Physician VAS (0–100)	36.0 (24.0, 60.0)
Positive ANA, *n* = 156	76 (48.7)
Positive HLA-B27, *n* = 135	20 (14.8)
Positive RF, *n* = 149	8 (5.4)
Positive Anti-CCP, *n* = 81	6 (7.4)
CRP mg/dL, *n* = 156	1.7 (1.0, 9.6)
ESR mm/hr., *n* = 151	11.0 (5.0, 32.0)

*ANA* Anti-nuclear antibody, *Anti-CCP* Anti-cyclic citrullinated peptide, *CRP* C-reactive protein, *ESR* Erythrocyte sedimentation rate, *HLA-B27* Human Leucocyte Antigen-B27, *Km* Kilometers, *RF* Rheumatoid factor, *VAS* Visual analog scale

^a^Active joints include joints which are swollen joints AND joints with pain and limited range of motion not caused for other reasons. This applies to joints including hips, temporomandibular joints where you cannot see swelling easily on exam

### Adherence to WT benchmarks by JIA category

Table 2 shows the distribution of JIA categories, the median WTs (IQR) and the percentage in each category meeting the WT benchmarks. Overall the median WT was 22 days between referral and pediatric rheumatologist visit (IQR 9, 45), and there was no statistically significant difference in WTs among JIA categories (*p* = 0.055). The majority of sJIA cases met the 7-day benchmark (67%), with a median WT of 1.5 days (0, 29). High rates of benchmark adherence were also seen in patients with RF-positive JIA (71%) and oligoarticular JIA (69%). Although fewer patients with psoriatic JIA (38%) and enthesitis-related arthritis (45%) met the 4-week benchmark, the WTs for these categories were not statistically significantly different from other JIA categories, although sample sizes were small, potentially limiting statistical significance. Overall, 62% of JIA cases were seen within the established WT benchmarks. The WTs were also evaluated based on calendar year and no obvious linear trend was noted.

**Table 2:** JIA category and percent meeting wait time benchmarks

JIA Category	N (% of total cohort)	Median WT d (IQR)	N (% meeting WT benchmark)
Enthesitis-related JIA	20 (12.2)	41.5 (13, 121)	9/20 (45%)
Oligoarticular JIA	75 (45.7)	19.0 (9.0, 40)	52/75 (69.3%)
Psoriatic JIA	8 (4.9)	40.5 (11.5, 55.0)	3/8 (37.5%)
RF negative JIA	48 (29.3)	23.0 (13.0, 39.5)	28/48 (58.3%)
RF positive JIA	7 (4.3)	6 (2.0, 91.0)	5/7 (71.4%)
Systemic JIA	6 (3.7)	1.5 (0.0, 29.0)	4/6 (66.7%)

Benchmarks for Systemic JIA are 7 days and for all other JIA categories are 4 weeks (28 days)

*d* Days, *IQR* Interquartile range, *JIA* Juvenile idiopathic arthritis, *RF* Rheumatoid factor

### Factors associated with meeting WT benchmarks

Six observations were removed from the Cox proportional hazards modeling as the referral data and visit date occurred on the same date, indicating these patients were not triaged according to the usual methods (i.e., were seen as urgent on-call cases) leaving 158 observations for the analysis. In the base model, which included age, sex, network distance, number of active joints, baseline pain VAS score and CRP, higher age was associated with longer WTs (HR 0.94, 95% CI 0.89, 0.98, *p* = 0.005). No other variables were statistically associated with WTs (Table 3). In a sensitivity analysis using JIA category, age, sex, pain and network distance, age at diagnosis remained as the only significant variable (HR 0.94, 95% CI 0.89, 0.98, *p* = 0.01).

**Table 3:** Cox proportional hazard modeling of JIA wait times

Variables included in the model	Hazard Ratio (95% CI)	*P* value
Age at diagnosis	0.94 (0.89, 0.98)	**0.005**
Female sex	0.73 (0.47, 1.13)	0.16
Network distance in Km	1.00 (1.00, 1.00)	0.17
Number of Active Joints	0.99 (0.95, 1.02)	0.45
Pain (VAS)	1.00 (0.99, 1.00)	0.53
CRP	1.01 (1.00, 1.02)	0.27

Network distance: shortest distance between patient residence and the pediatric rheumatology center along roadways

*CRP* C-reactive protein, *Km* Kilometers, *VAS* Visual analog scale

## Discussion

While the median WTs for patients with JIA in this study met the national benchmarks, 38% of patients still experienced longer WTs for appointments indicating additional work needs to be done to improve access to pediatric rheumatology care. WTs did not significantly differ by JIA category or distance to the rheumatology center, but older patients waited longer.

Longer waits for older JIA patients were also observed in a French study, as were joint pain and the presence of enthesitis, while abnormal inflammatory markers and joint swelling or limp were associated with shorter WTs [10]. It is possible that younger patients with oligoarthritis present more frequently with knee arthritis and limping, which is readily identified. Older children may also be less likely to bring non-painful swollen joints to the attention of family and healthcare providers.

Interestingly, our study demonstrated similar adherence to WT benchmarks as a UK study of 10 pediatric rheumatology centers measured against the British Society for Paediatric and Adolescent Rheumatology/Arthritis and Musculoskeletal Alliance Standards of Care (BSPAR/ARMA) for JIA, which also set a 4 week benchmark [11]. In that study 60% of JIA cases were seen within the benchmark; however, factors associated with delays were not identified [11]. A 2009 German study of predictors of delayed referral to pediatric rheumatology revealed much longer median WTs to pediatric rheumatologist visit (90 days, range 0–2160 days). The predictors of delayed referral included referrals from orthopedics and greater distance of the patient’s residence to the pediatric rheumatology center [12]. The Research on Arthritis in Canadian Children Emphasizing Outcomes (ReACCh Out) study evaluated factors associated with longer time between symptom onset and the first pediatric rheumatology visit in 319 children enrolled between 2005 and 2007. The median duration between symptom onset to first visit was 115 days (IQR 45, 219) [13], highlighting that there may be delays not under the control of the pediatric rheumatology clinic. Factors associated with shorter duration of time between symptom onset and rheumatology visit included fever, being of South Asian ethnicity, or having a limp, while a history of heel pain or enthesitis was associated with a longer time to assessment. In our study we also considered whether an abnormal CRP would be associated with a shorter WT, as although nonspecific, high CRP can be associated with higher disease activity prompting more urgent assessment, although we did not find an association with WTs.

This study represents the first time that WTs for JIA have been systematically evaluated in our center, and limitations include the use of data from an existing pharmacogenetics study to evaluate WTs, rather than all patients with JIA seen at our center (as this data is not routinely collected), resulting in the possibility of selection bias. Additionally, amongst pharmacogenetics study participants, almost half of referral dates were not available in the chart. However, the JIA category distribution in this study reflects the expected distribution of the JIA population in our center and indeed in Canada. The numbers of patients with some categories of JIA were small, which may have limited our statistical power. For example, WTs in psoriatic JIA and enthesitis-related categories approached statistical significance and it is possible with a larger sample that these may have reached statistical significance. We did not extract data from the patients’ referrals, so the relationship between the factors associated with WTs is based on patient characteristics at the baseline rheumatology visit, rather than at time of referral. We also did not have information about symptom onset, which may be important to measure, as patients may wait a long time before a referral is sent to the pediatric rheumatology center. We also lacked information on parental education and household income which are often important in studies on access to care and a majority of our patients were Caucasian which limited meaningful analysis of wait times by ethnicity.

## Conclusion

In conclusion, WTs are important system level measures of quality of care. In future, additional measures including monitoring the number of pediatric rheumatology visits per year (new and follow-up), total number of referrals for JIA, number of pediatric rheumatologists, trainees and other allied healthcare providers may be important to track to better understand factors influencing WT measures. Such measures could be tracked when implementing interventions to decrease WTs and improve care. Although this study shows 62% adherence to national WT benchmarks for time between referral to and assessment by pediatric rheumatology, routine data collection about WTs that includes details about patient diagnosis, information contained within referrals, and symptoms at onset, would help identify and rectify gaps in the timely provision of care. Future work is planned nationally to better track performance measures including WTs in JIA.

## Data Availability

The datasets generated and/or analysed during the current study are not publicly available as they contain identifiable information (i.e. postal codes).

## Abbreviations

BSPAR/ARMA: British Society for Paediatric and Adolescent Rheumatology/Arthritis and Musculoskeletal Alliance
CI: Confidence interval
CRP: C-reactive protein
HR: Hazard ratio
ILAR: International League of Associations for Rheumatology
IQR: Inter Quartile Range
JIA: Juvenile Idiopathic Arthritis
Km: Kilometer
ReACCh Out: Research on Arthritis in Canadian Children Emphasizing Outcomes
RF: Rheumatoid factor
sJIA: Systemic Juvenile Idiopathic Arthritis
VAS: Visual analog scale
WT: Wait times
